# A Story of Science

**Published:** 1887-08

**Authors:** 


### A Story of Science.

By One Who Knows Nothing About It.

A philosopher sat in his easy chair,
  Looking as grave as Milton;
He wore a solemn and mystic air
  As he Canada balsam spilt on
A strip of glass, a slide to prepare
  For a mite taken out of his Stilton.
He took his microscope out of its case,
  And settled the focus rightly;
The light thrown back from the mirror's face
  Came glimmering upward brightly.
He put the slide with the mite in place,
  And fixed on the cover tightly.
He turned the instrument up and down,
  Till getting a proper sight, he
Exclaimed, as he gazed with a puzzled frown,
  "Good gracious!" and "Highty=tighty!
The sight is enough to alarm the town—
  A mite is a monster mighty!"
From 'tother end of the tube, the mite
  Regarded our scientific,—
To his naked eye, as you'll guess, the sight
  Of a man was most terrific,
But reversing the microscope, made him quite
  The opposite of magnific.
"One sees the truth through this tube so tall,"
  Said the mite as he squinted through it,
"Man is not so wondrously big after all,
    If the mite-world only knew it."

Moral.

Mem.—Whether a thing is large or small
  Depends on the way you view it!

